# The new face of dialysis: aging, diabetes, and challenges for vascular access

**DOI:** 10.1590/2175-8239-JBN-2026-E004en

**Published:** 2026-02-16

**Authors:** Rosilene M. Elias, Benedito Jorge Pereira, Maria Eugenia F. Canziani

**Affiliations:** 1 Universidade de São Paulo, Hospital das Clínicas, Departamento de Medicina, Divisão de Nefrologia, São Paulo, SP, Brazil.; 2 Universidade Nove de Julho, São Paulo, SP, Brazil.; 3 Universidade Federal de São Paulo, Escola Paulista de Medicina, Departamento de Medicina – Nefrologia, São Paulo, SP, Brazil.

## Introduction

The Brazilian Dialysis Census, created in the 2000s, is a crucial source of information on dialysis treatment in Brazil, carefully prepared by the Brazilian Society of Nephrology. Although the response rate from dialysis centers is incomplete and the surveys are subject to biases inherent to the method, real-world data remain essential for understanding clinical practice. These records complement evidence from clinical trials by capturing real variations in care and outcomes in heterogeneous populations. Recent studies reinforce the value of this type of registry, demonstrating that, despite its limitations, real-world data constitute an indispensable pillar for interpreting the healthcare landscape. Using data obtained from the Census, it is possible to outline an epidemiological profile and, based on this, inform society and promote strategies to improve care for patients with chronic kidney disease (CKD).

The scenario of kidney replacement therapy in Brazil has undergone a notable transformation in recent decades. Data from the 2024 Dialysis Census^1^ point to three trends: (1) a significant increase in diabetes mellitus as the etiology of CKD among dialysis patients, reaching the same proportion as arterial hypertension, which until then had been the leading cause; (2) the progressive aging of the dialysis population, with more than one-third of patients over 65 years old (37.8% of cases); and (3) an increase in the use of long-term central venous catheters (23%) and a persistently high rate of non-tunneled catheters (8%). These changes reflect not only epidemiological shifts but also structural and care-related weaknesses within nephrology services.

## Change in Epidemiological Profile

CKD has become a complication of aging and diabetes, bringing significant clinical challenges: older patients with multiple comorbidities, which directly affect prognosis and quality of life.

The increase in patients with diabetes, especially among older adults, reinforces the need for integrated approaches involving nephrology, geriatrics, cardiology, and endocrinology, with a focus on prevention, education, and individualized management of metabolic and cardiovascular risk.

## The Dilemma of Vascular Access

In parallel, there has been an increase in the use of central venous catheters as vascular access. This trend, although understandable in the context of an aging population and increasingly complex patients, suggests a step backward in terms of safety and quality of care. This is because catheters are associated with higher risks of infection, hospitalization, and mortality, as well as increased costs^2^. The decline in the use of arteriovenous fistulas (AVFs), particularly among older adults, proposes the need for early planning of kidney replacement therapy and better coordination between nephrologists and vascular surgeons. Although the primary patency of AVFs is lower in older individuals, secondary patency is similar^3^. The survival of older patients on maintenance dialysis depends on factors such as comorbidities, race, and functional status^4,5^. Thus, it can be considered that, in patients with a lower mortality risk, the AVF remains the best option, whereas continuous catheter use becomes a relevant risk factor, reinforcing the need for individualized decision-making. However, it should be noted that the guidelines published in the most recent KDOQI (Kidney Disease Outcomes Quality Initiative) emphasize the concept of the “life plan,” in which the catheter may, at times, be used according to individual preferences and specific patient conditions—certainly the case for many older patients^6^.

There is particular concern regarding the use of temporary catheters. The 2024 Census^1^ shows that 8% of patients use a temporary catheter. This represents 13,800 patients, a non-negligible number of individuals at higher risk of infection. It is time-critical that actions be developed to change this scenario so that the use of this type of access is once again regarded as temporary. Becoming complacent and holding the false impression that a temporary catheter can be used for a prolonged period cannot be considered a safe practice free of complications. The nephrology community must come together to change this situation, since the literature does not recommend the use of this type of access for more than 2-3 weeks^6,7,8^, mainly due to the increased risk of infection.

## Implications and Opportunities

These trends require a strategic revision of CKD care, beginning in the pre-dialysis phase. It is urgent to invest in:

- Early detection programs and intensive control of diabetes and hypertension, particularly through training multidisciplinary teams and strengthening primary care.
- A planned transition to dialysis, with early evaluation of vascular access.
- Centers with care models designed for older individuals that prioritize functionality, quality of life, and shared decision-making.

## CONCLUSION

The dialysis census is not merely a statistical snapshot; it is an epidemiological and ethical alert. The increase in average age, diabetes prevalence, and catheter use should prompt a rethinking of the model of care, with greater emphasis on integration, prevention, and planning.

Focusing solely on the technical survival of dialysis is insufficient. It is time to place the patient, not the procedure, at the center of decision-making.

The still modest increase in the use of peritoneal dialysis in the country (5.6%) is extremely positive, as this modality is associated with better quality of life and should not be neglected in the face of technological advances. Within this context of innovation, it is notable that 7.1% of the dialysis population already undergoes hemodiafiltration, currently limited to privately funded systems. Home hemodialysis, in turn, is beginning to appear in the Census and is expected to expand in the coming years, increasing patient access both by allowing a higher number of treatments outside dialysis centers and by freeing up slots within them.

The new face of dialysis reflects the aging of society and the extent to which we, too, must “age” in terms of clinical maturity and public policy.

## Data Availability

No data were associated with this editorial.
