# Supplementary material for: The new face of dialysis: aging, diabetes, and challenges for vascular access
Source: J Bras Nefrol. 2026 Feb 16;48(1):e2026E004. doi: 10.1590/2175-8239-JBN-2026-E004en (PMC12927271; doi:10.1590/2175-8239-JBN-2026-E004en)
Supplement: Translation

# Translation: O novo rosto da diálise: envelhecimento, diabetes e desafios para o acesso vascular

**DOI:** 10.1590/2175-8239-JBN-2026-E004pt

**Authors:** Rosilene M. Elias, Benedito Jorge Pereira, Maria Eugenia F. Canziani

**Affiliations:** 1 Universidade de São Paulo, Hospital das Clínicas, Departamento de Medicina, Divisão de Nefrologia, São Paulo, SP, Brasil.; 2 Universidade Nove de Julho, São Paulo, SP, Brasil.; 3 Universidade Federal de São Paulo, Escola Paulista de Medicina, Departamento de Medicina – Nefrologia, São Paulo, SP, Brasil.

## Introdução

O Censo Brasileiro de Diálise, criado nos anos 2000, é uma fonte crucial de informação sobre o tratamento dialítico no país, preparado com zelo pela Sociedade Brasileira de Nefrologia. Embora a taxa de resposta dos centros de diálise não seja completa e os inquéritos estejam sujeitos a vieses inerentes ao método, os dados de mundo real permanecem essenciais para compreender a prática assistencial. Esses registros complementam as evidências dos ensaios clínicos ao capturar variações reais de cuidado e desfechos em populações heterogêneas. Estudos recentes reforçam o valor desse tipo de registro, demonstrando que, apesar das limitações, os dados de mundo real constituem um pilar indispensável para interpretar o cenário assistencial. Com os dados obtidos do Censo, é possível traçar um perfil epidemiológico e, a partir dele, informar a sociedade e promover estratégias de melhoria no cuidado ao paciente com Doença Renal Crônica (DRC).

O cenário da terapia renal substitutiva no Brasil demonstra uma transformação marcante nas últimas décadas. Dados do Censo de Diálise 2024^1^ apontam três tendências principais: (1) o aumento expressivo do diabetes mellitus como etiologia da DRC em diálise, igualandose à hipertensão arterial, que até então se configurava como principal etiologia; (2) o envelhecimento progressivo da população em diálise, com mais de um terço dos pacientes com idade < 65 anos (37,8% dos casos); e (3) o aumento do uso de cateter venoso central de longa permanência (23%) e a mantida taxa de 8% de cateteres não tunelizados. Essas mudanças refletem não apenas transformações epidemiológicas, mas também fragilidades estruturais e assistenciais do cuidado nefrológico.

## Mudança no Perfil Epidemiológico

A DRC tornou-se uma complicação do envelhecimento e do diabetes, trazendo desafios clínicos significativos: pacientes mais velhos e com múltiplas comorbidades, o que impacta diretamente o prognóstico e a qualidade de vida.

O aumento do número de pacientes com diabetes, sobretudo entre os mais velhos, reforça a necessidade de abordagens integradas entre a nefrologia, a geriatria, a cardiologia e a endocrinologia, com foco na prevenção, na educação e no manejo individualizado do risco metabólico e cardiovascular.

## O Dilema do Acesso Vascular

Paralelamente, observa-se o crescimento do uso de cateteres venosos centrais como acesso vascular. Essa tendência, embora compreensível diante do envelhecimento e da maior complexidade dos pacientes, sugere um retrocesso em termos de segurança e qualidade assistencial. Isso se deve ao fato de o cateter associar-se a maior risco de infecção, internação e mortalidade, além decustos maiselevados^2^. A queda no uso de fístulas arteriovenosas (FAV), especialmente em idosos, sugere a necessidade de planejamento precoce da terapia renal substitutiva e melhor coordenação entre nefrologistas e cirurgiões vasculares. Embora a patência primária das FAVs seja menor em pessoas idosas, a patência secundária é similar^3^. A sobrevida dos pacientes idosos em diálise depende de fatores como comorbidades, raça e funcionalidade^4,5^. Dessa forma, é possível considerar que, em pacientes com menor risco de mortalidade, a FAV ainda é a melhor opção, enquanto o uso contínuo do cateter se torna um fator de risco relevante, reforçando a necessidade de decisões individualizadas. Entretanto, deve-se considerar que as diretrizes publicadas na mais recente atualização do KDOQI (*Kidney Disease Outcomes Quality Initiative*) nos ensinam sobre o plano de vida, no qual, muitas vezes, o cateter pode ser utilizado de acordo com as preferências individuais e as condições específicas dos pacientes, o que certamente se aplica aos pacientes mais idosos^6^.

Ressalta-se, porém, a preocupação com o uso de cateter temporário. O Censo de Diálise 2024^1^ mostra que 8% dos pacientes utilizam um cateter temporário. Isso representa cerca de 13.800 pacientes, um número não desprezível de indivíduos sob maior risco de infecção. É urgente que se desenvolvam ações no sentido de mudar esse cenário, para que o uso desse tipo de acesso volte a ser considerado, de fato, temporário. Acomodar-se e manter a falsa impressão de que um cateter temporário possa ser usado por tempo prolongado não pode ser encarado como prática segura e isenta de complicações. A comunidade nefrológica precisa se unir para transformar esse cenário, uma vez que a literatura não recomenda o uso desse tipo de acesso por período superior a 2-3 semanas^6,7,8^, principalmente em razão do maior risco de infecção.

## Implicações e Oportunidades

Essas tendências exigem uma revisão estratégica do cuidado em DRC, desde a fase pré-diálise. É urgente investir em:

- Programas de detecção precoce e controle intensivo do diabetes e da hipertensão, especialmente por meio da capacitação de equipes multidisciplinares e do fortalecimento da atenção primária.
- Transição planejada para diálise, com avaliação antecipada do acesso vascular.
- Modelos de cuidado centrados no idoso, que priorizem funcionalidade, qualidade de vida e decisões compartilhadas.

## Conclusão

O Censo de Diálise não é apenas um retrato estatístico; é um alerta epidemiológico e ético. O aumento da idade média, do diabetes e do uso de cateteres deve nos levar a repensar o modelo de cuidado, buscando maior integração, prevenção e planejamento.

Manter o foco somente na sobrevida técnica da diálise é insuficiente. É hora de colocar o paciente — e não o procedimento — no centro das decisões.

O aumento ainda discreto, no país, da utilização da diálise peritoneal (5,6%) é extremamente positivo, uma vez que essa modalidade está associada a melhor qualidade de vida e não deve ser negligenciada diante dos avanços tecnológicos. No contexto dessas inovações, observa-se que 7,1% da população em diálise já realiza hemodiafiltração, atualmente limitada aos sistemas privados de financiamento. A hemodiálise domiciliar, por sua vez, começa a surgir no Censo e tende a expandir-se nos próximos anos, ampliando a acessibilidade aos pacientes, tanto por permitir maior número de atendimentos fora dos centros quanto por liberar vagas nesses.

O novo rosto da diálise reflete o envelhecimento da sociedade e evidencia o quanto precisamos envelhecer também em termos de maturidade clínica e de políticas públicas.

## Data Availability

Nenhum dado foi associado ao presente editorial.
